# Supplementary material for: Bactericidal membrane attack complex formation initiates at the new pole of E. coli
Source: EMBO Rep. 2025 Dec 8;27(2):533–54. doi: 10.1038/s44319-025-00669-1 (PMC12852941; doi:10.1038/s44319-025-00669-1)
Supplement: Supplementary file 1 — Appendix [file 44319_2025_669_MOESM1_ESM.pdf]

Appendix

Bactericidal Membrane Attack Complex formation initiates at the new pole of *E. coli*

Marije F.L. van 't Wout, Fabian Hauser, Philippa I.P. Holzapfel, Bart W. Bardoel, Carla J.C. de Haas, Jaroslaw Jacak, Suzan H.M. Rooijakkers and Dani A.C. Heesterbeek

Table of Contents

Appendix Figure S1 - Gating strategy for flow cytometry graphs 2

Appendix Figure S2 - Flow cytometry and overview image of MAC deposition on *E. coli* MG1655........ 3

Appendix Figure S3 - Overview image analysis pipeline of bacteria 5

Appendix Figure S4 - Training and evaluation of bacterial object detection and segmentation models 7

Appendix Figure S5 - Simulations for the image analysis pipeline 8

Appendix Figure S6 - Flow cytometry and overview image of MAC deposition on *E. coli* EC10 10

Appendix Figure S7 - Flow cytometry and overview image of MAC deposition on *E. coli* MG1655 after antibody-mediated complement activation 11

Appendix Figure S8 - Distribution of HADA-labeled peptidoglycan over time 12

Appendix Figure S9 - Classification of bacteria at the start of time-lapse imaging 13

Appendix Figure S10 - Linear regression analysis of C9 intensity, Sytox intensity and bacterial growth after MAC deposition 14

Appendix Figure S11 - Comparison of channel swapping 16

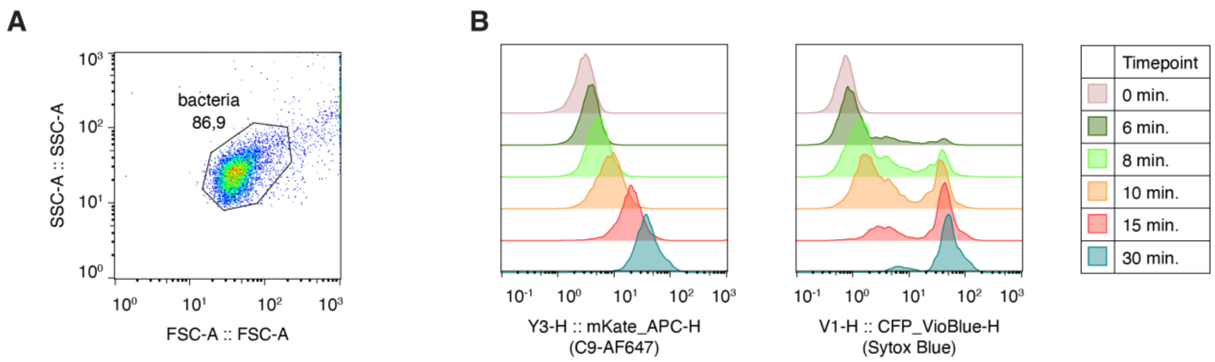

Appendix Figure S1 - Gating strategy for flow cytometry graphs

A Gating strategy to gate bacteria based on forward and side scatter.

B Histograms showing the fluorescence intensity profiles of C9-AF647 and Sytox Blue (height values).

Data information: Example shows one biological replicate of figure 1B.

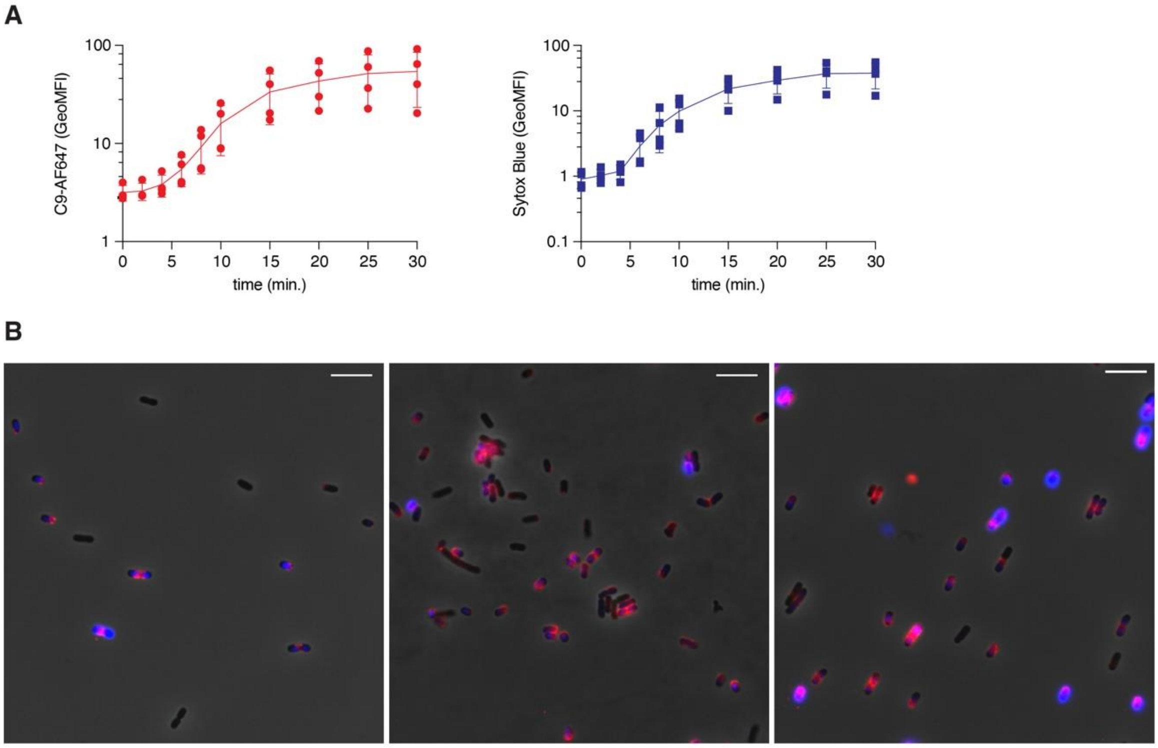

Appendix Figure S2 - Flow cytometry and overview image of MAC deposition on *E. coli* MG1655

A GeoMFI values for C9-AF647 deposition and inner membrane damage (Sytox Blue) before normalization (Fig. 1B).

B Example images of bacteria that were exposed to MAC components for 8 minutes, showing results of three different biological replicates. C9-AF647 is shown in red and Sytox in blue.

Data information: In (A), data represent individual values with mean ± SD of four biological replicates. In (B), scale bars: 10 µm.

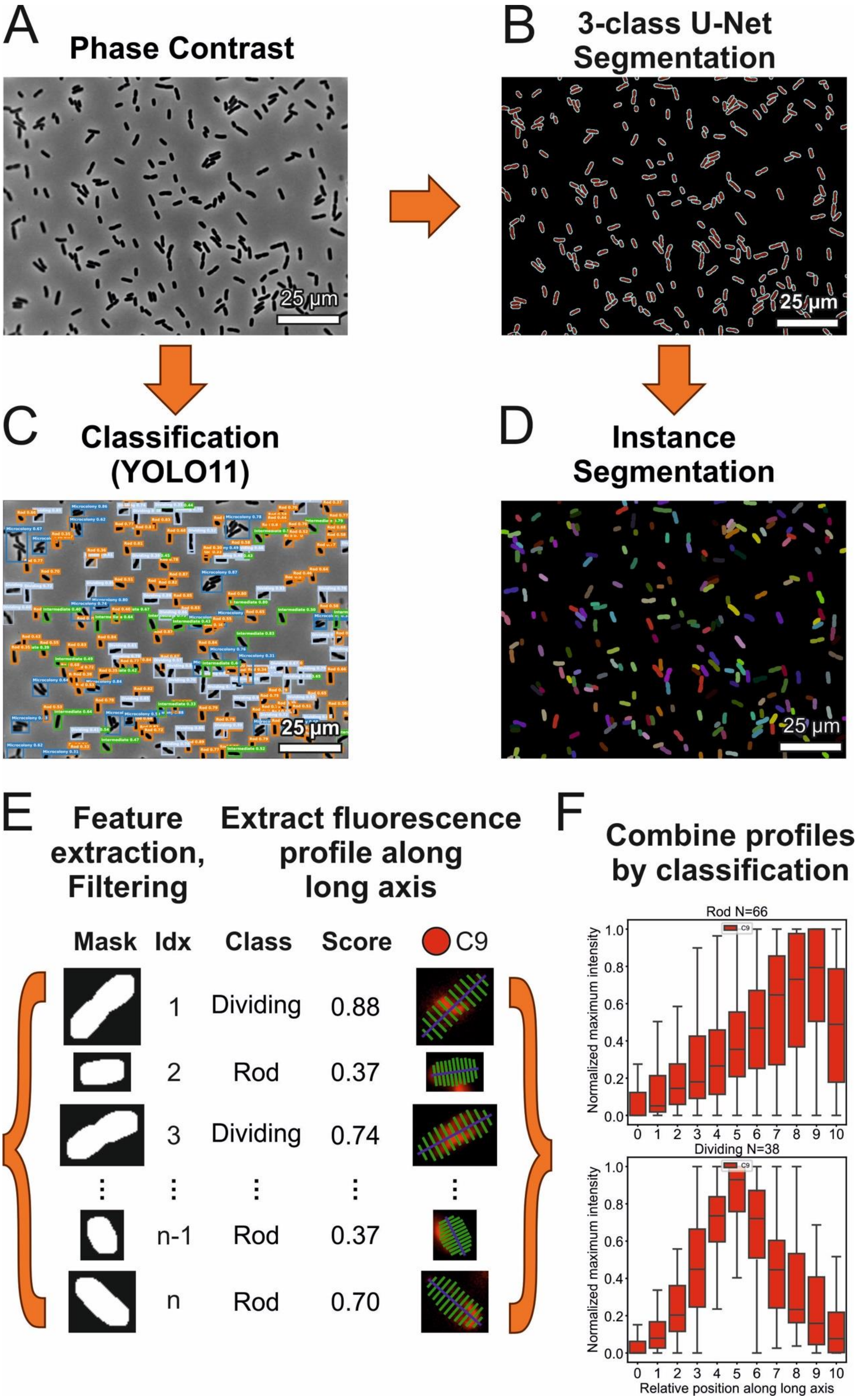
A
Phase Contrast
25 µm
B
3-class U-Net Segmentation
25 µm
C
Classification (YOLO11)
25 µm
D
Instance Segmentation
25 µm
E
Feature extraction, Filtering
Extract fluorescence profile along long axis
Mask
Idx
Class
Score
C9
1
Dividing
0.88
2
Rod
0.37
3
Dividing
0.74
n-1
Rod
0.37
n
Rod
0.70
F
Combine profiles by classification
Rod N=66
Dividing N=38
Normalized maximum intensity
Relative position along long axis

Appendix Figure S3 - Overview image analysis pipeline of bacteria

A An exemplary phase-contrast microscopy image of Gram-negative bacteria.

B Semantic segmentation results from a trained 3-class U-Net which predicted bacterial masks for the background, cell membrane and cell body from (A).

C The object classification results predicted from another custom trained deep learning model (YOLO11) are visualized. Thereby bounding boxes, object classification into bacterial growth stage (microcolonies, dividing, rod shaped, intermediate and defocused bacteria) and classification scores were predicted from the same phase-contrast image as in (A).

D False color instance segmentation masks of individual cells based on the 3-class U-Net predictions from (B). Each color represents a single cell with a unique number and mask associated.

E The pipeline for processing each instance segmented cell. In this step, cells and their associated mask (cell 1 … n) were further refined. Each cell was then correlated with a matching YOLO result by highest intersection over union value and classification confidence score. Outliers were automatically filtered (i.e. undesired growth stages). Next the geometry of cells is further processed and based on the cell mask the long axis (blue line) of the bacteria is determined by the image momentum algorithm (see Material & Methods chapter “Image processing pipeline and fluorescence intensity profile calculation” for details). The long axis (blue line) was then divided equidistantly into N = 11 points. A fluorescence intensity profile along the long axis was calculated by selecting the maximum pixel value sampled (bilinear interpolation) along the perpendicular lines (green lines). This was repeated for each fluorescent channel and cell.

F All calculated fluorescence signal profiles along the long axis of individual bacteria split by their growth stage (rod and dividing) in two box plots.

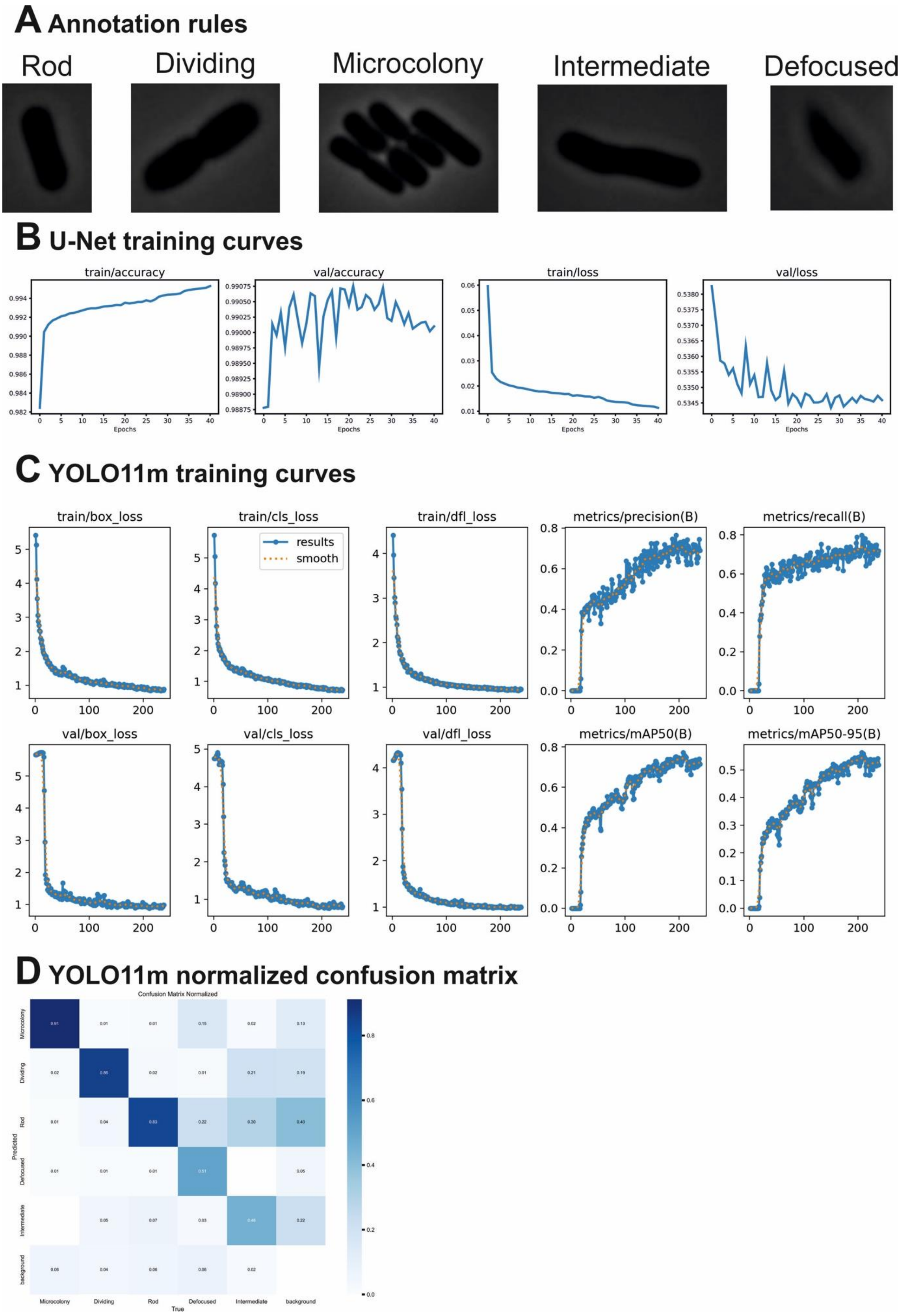
A Annotation rules
Rod
Dividing
Microcolony
Intermediate
Defocused
B U-Net training curves
train/accuracy
val/accuracy
train/loss
val/loss
Epochs
C YOLO11m training curves
train/box_loss
train/cls_loss
train/dfl_loss
metrics/precision(B)
metrics/recall(B)
results
smooth
val/box_loss
val/cls_loss
val/dfl_loss
metrics/mAP50(B)
metrics/mAP50-95(B)
D YOLO11m normalized confusion matrix
Confusion Matrix Normalized
Predicted
True
Microcolony
Dividing
Rod
Defocused
Intermediate
background

Appendix Figure S4 - Training and evaluation of bacterial object detection and segmentation models

A Annotation rules for YOLO training, with example images for each class: Early/rod-shaped (recently divided, very small rods), Middle/intermediate (elongated compared to early stage but without a septum), Late/dividing (showing a clear septum or indentation at midcell), and Microcolony (cells attached to one or more other bacteria).

B U-Net training curves over 41 epochs, showing accuracy and loss for training and validation data.

C YOLOv11m training curves over 239 epochs, showing precision, recall, mAP50, mAP50–95, and training/validation metrics for box loss, classification loss, and DFL loss.

D YOLOv11m normalized confusion matrix with classes: Microcolony, Dividing, Rod, Defocused, Intermediate, Background. The most important classes, Dividing and Rod, were classified with high accuracy (86% and 83%, respectively).

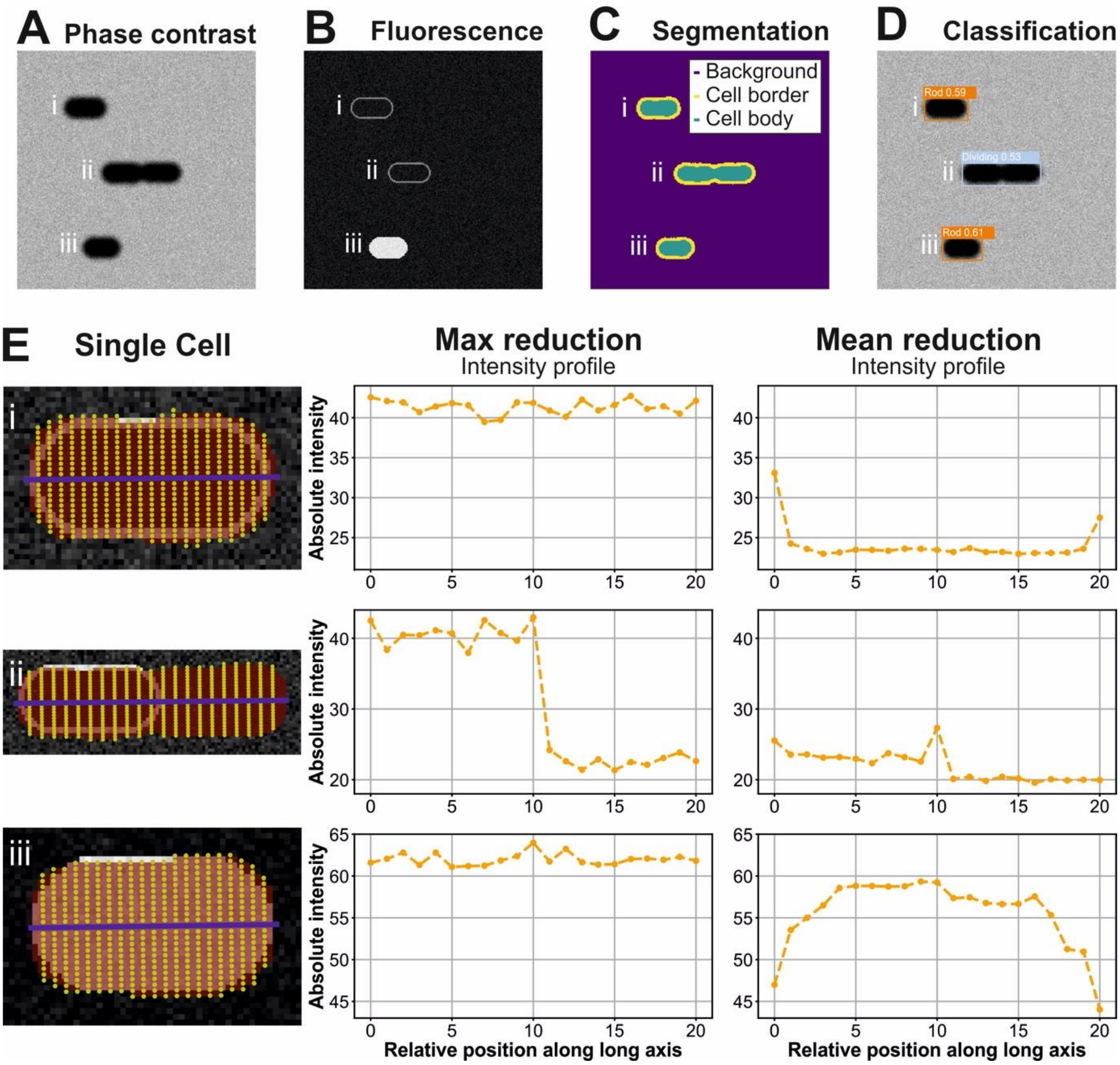

Appendix Figure S5 - Simulations for the image analysis pipeline

A Simulation of phase-contrast microscopy of three bacteria cells at two different growth stages (i+iii: rod shaped, ii: dividing). Noise values (Poisson and Gaussian) were chosen to match the intensity levels of the experiments used in this study (unsigned 8-bit grayscale image with pixels values from 40 to 200).

B Simulation of fluorescent signals of the same three bacteria as in (A) resembling i: signals only in the membrane of a rod-shaped bacterium, ii: signals only in the membrane of a single sub-cell during division, and iii: signals in the whole cell with the same intensity. The pixel intensity values were again chosen to match experimental conditions (unsigned 8-bit grayscale image with pixels values from 10 to 66).

C Segmentation of the simulated phase contrast image from (A) using our trained deep neuronal network U-Net with 3 classes.

D YOLO object detection and classification of the simulated phase contrast image from (A) using our trained YOLO11 deep neuronal network.

E Application of our image analysis pipeline to all three simulated bacteria cells (one cell in each row). The first column shows the simulated fluorescence signal from (B) in the background of a single cells, in red the determined instance segmentation mask from (C), in blue the calculated long axis using the image momentum algorithm, and each yellow dot represents sampled pixels (bilinear interpolation) along a perpendicular line (to the long axis line) at equidistant points along the long axis line (N = 21). The second and third column show the calculated fluorescence intensity profile, where for each point along the long axis line (blue), the intensity values along the perpendicular line (yellow dots) were combined (reduced) by either the maximum value (second column) or the mean value (third column).

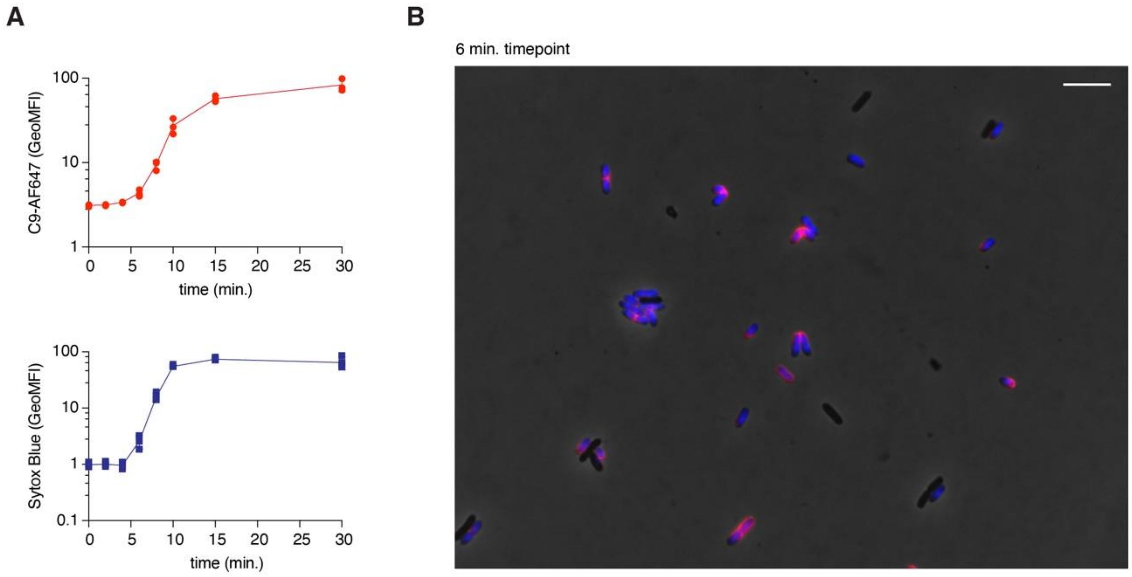

Appendix Figure S6 - Flow cytometry and overview image of MAC deposition on *E. coli* EC10

A GeoMFI values for C9-AF647 deposition and inner membrane damage (Sytox Blue) before normalization (Fig. EV1B).

B Example image of bacteria that were exposed to MAC components for 6 minutes. C9-AF647 is shown in red and Sytox in blue.

Data information: In (A), data represent individual values with mean ± SD of three biological replicates. In (B), scale bars: 10 µm.

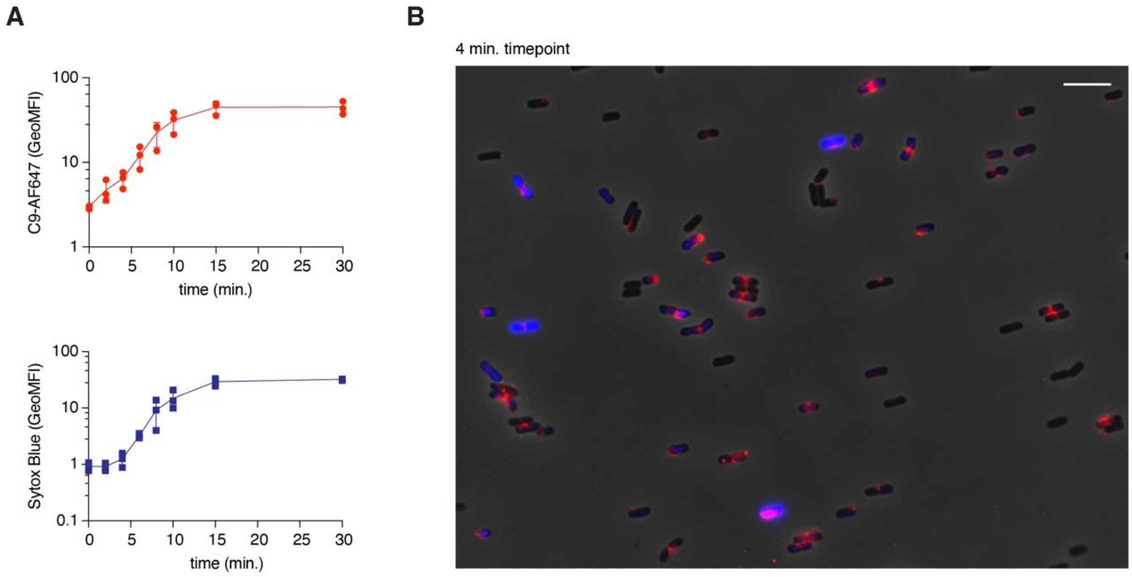

Appendix Figure S7 - Flow cytometry and overview image of MAC deposition on *E. coli* MG1655 after antibody-mediated complement activation

A GeoMFI values for C9-AF647 deposition and inner membrane damage (Sytox Blue) before normalization (Fig. 2B).

B Example image of bacteria that were exposed to MAC components for 4 minutes. C9-AF647 is shown in red and Sytox in blue.

Data information: In (A), data represent individual values with mean ± SD of three biological replicates. In (B), scale bars: 10 µm.

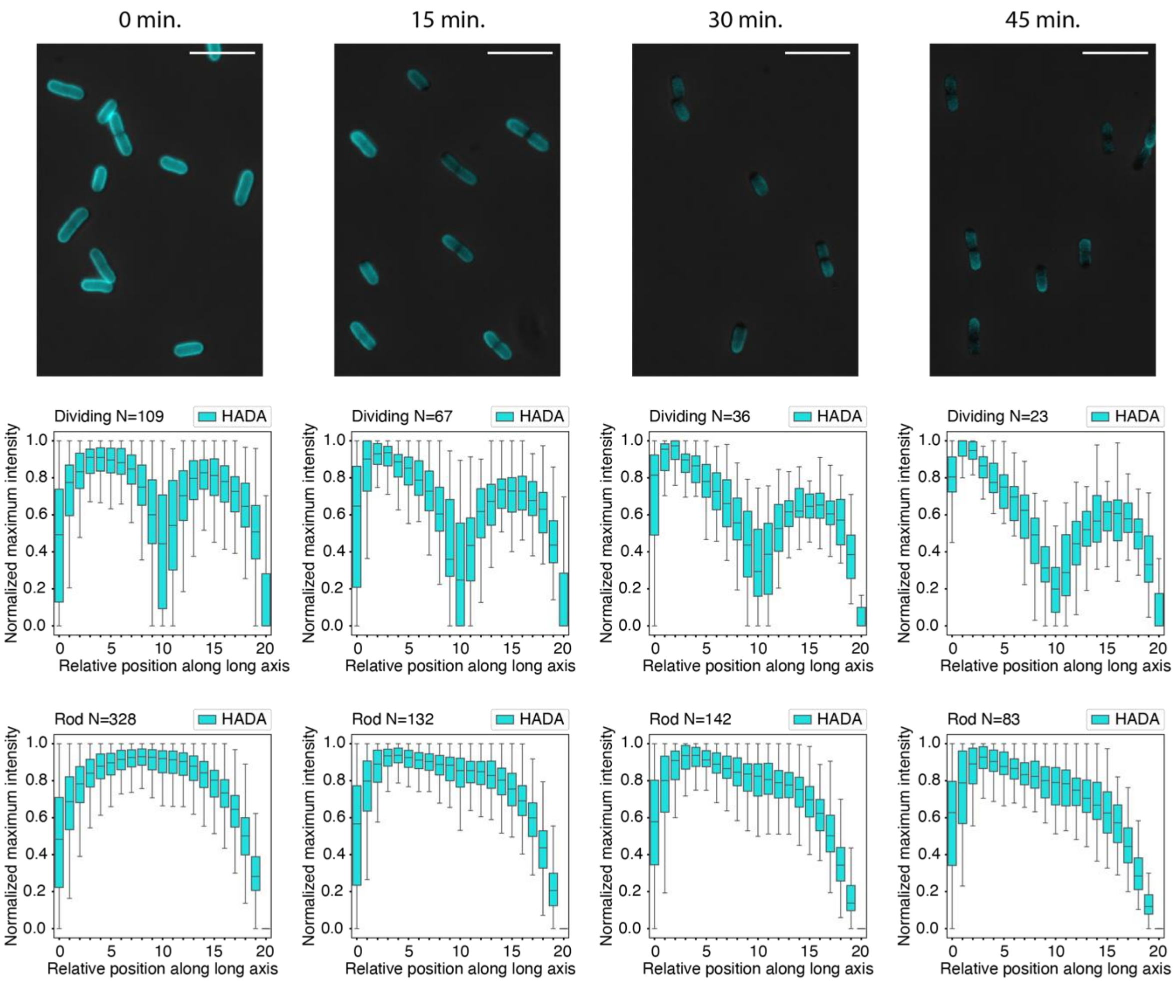

Appendix Figure S8 - Distribution of HADA-labeled peptidoglycan over time

After labeling the peptidoglycan of *E. coli* MG1655 with HADA, bacteria were allowed to grow in absence of HADA for up to 45 minutes. Samples were collected every 15 minutes and imaged by widefield fluorescence microscopy. Below each image, the normalized HADA distribution along the long axis of dividing and rod-shaped bacteria.

Data information: Data analysis was performed on all images that were taken at each timepoint and are representative for one biological replicate. In the box plots, each box represents the interquartile range (IQR) of the data, with the center line indicating the median relative intensity. The whiskers extend to the most extreme values within 1.5 x IQR. Scale bars: 10 µm.

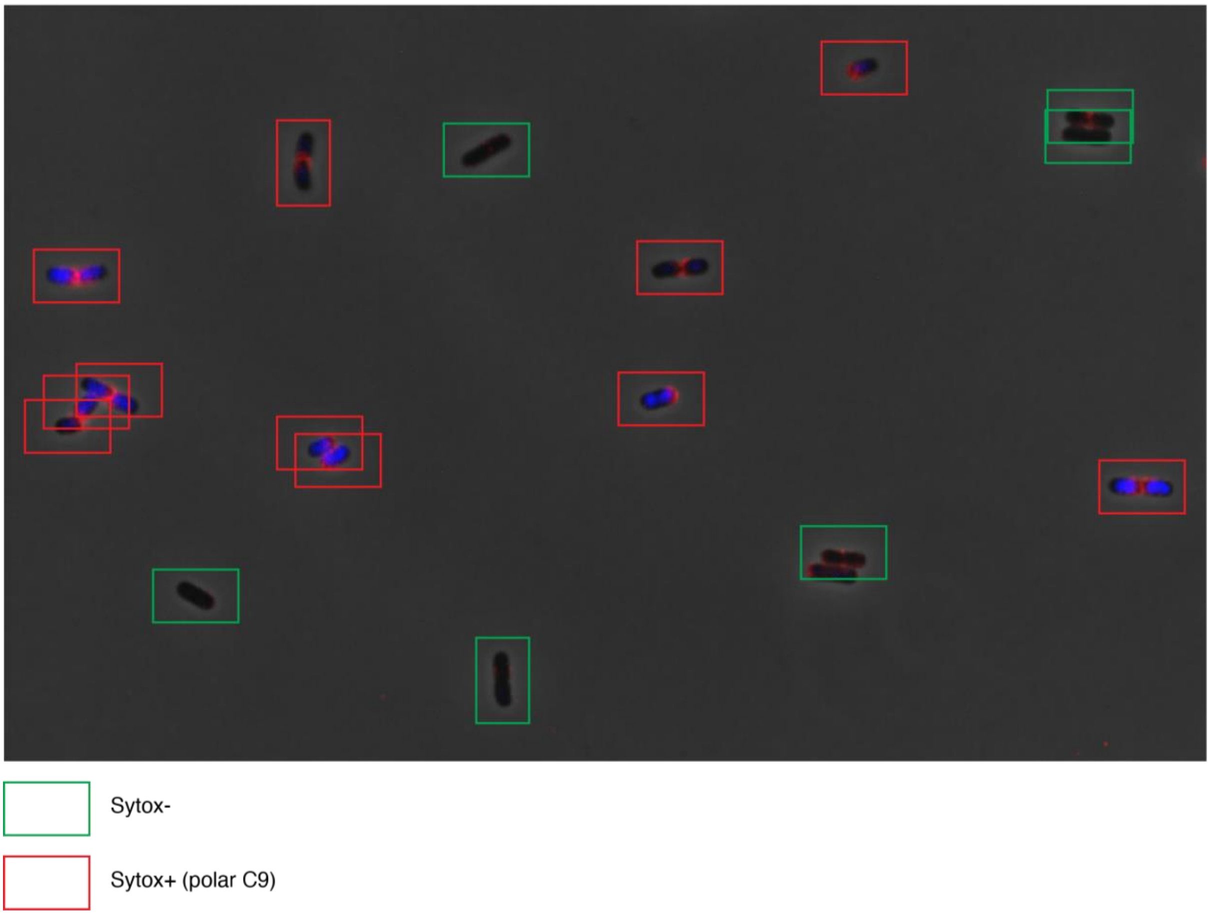

Appendix Figure S9 - Classification of bacteria at the start of time-lapse imaging

In this example, *E. coli* MG1655 had been pre-incubated with C5-depleted serum, washed, and then incubated with purified MAC components for 6 minutes (Fig. 4). The image shows bacteria at the start of time-lapse imaging. Bacteria were manually classified based on Sytox-positivity and MAC localization. Sytox-negative bacteria are annotated with green boxes and Sytox-positive bacteria with polar MAC localization with red boxes.

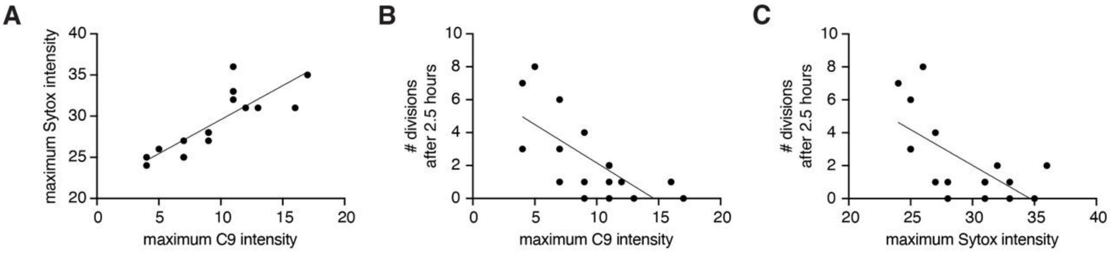

Appendix Figure S10 - Linear regression analysis of C9 intensity, Sytox intensity and bacterial growth after MAC deposition

A Linear regression of the maximum Sytox intensity against the maximum C9 intensity that was detected on the bacteria.

B Linear regression of the number of bacterial divisions against the maximum C9 intensity that was detected on the bacteria.

C Linear regression of the number of bacterial divisions against the maximum Sytox intensity that was detected on the bacteria.

Data information: Each point represents an individual bacterium after incubation with MAC components for 6 minutes (Fig. 4; Appendix Movie 3). The solid lines show the best-fit regression lines. In (A), slope = 0.83, R = 0.82, R^2 = 0.67, $p < 0.0001$. In (B), slope = -0.47, R = -0.70, R^2 = 0.48, p = 0.0019. In (C), slope = -0.43, R = -0.65, R^2 = 0.42, p = 0.0047.

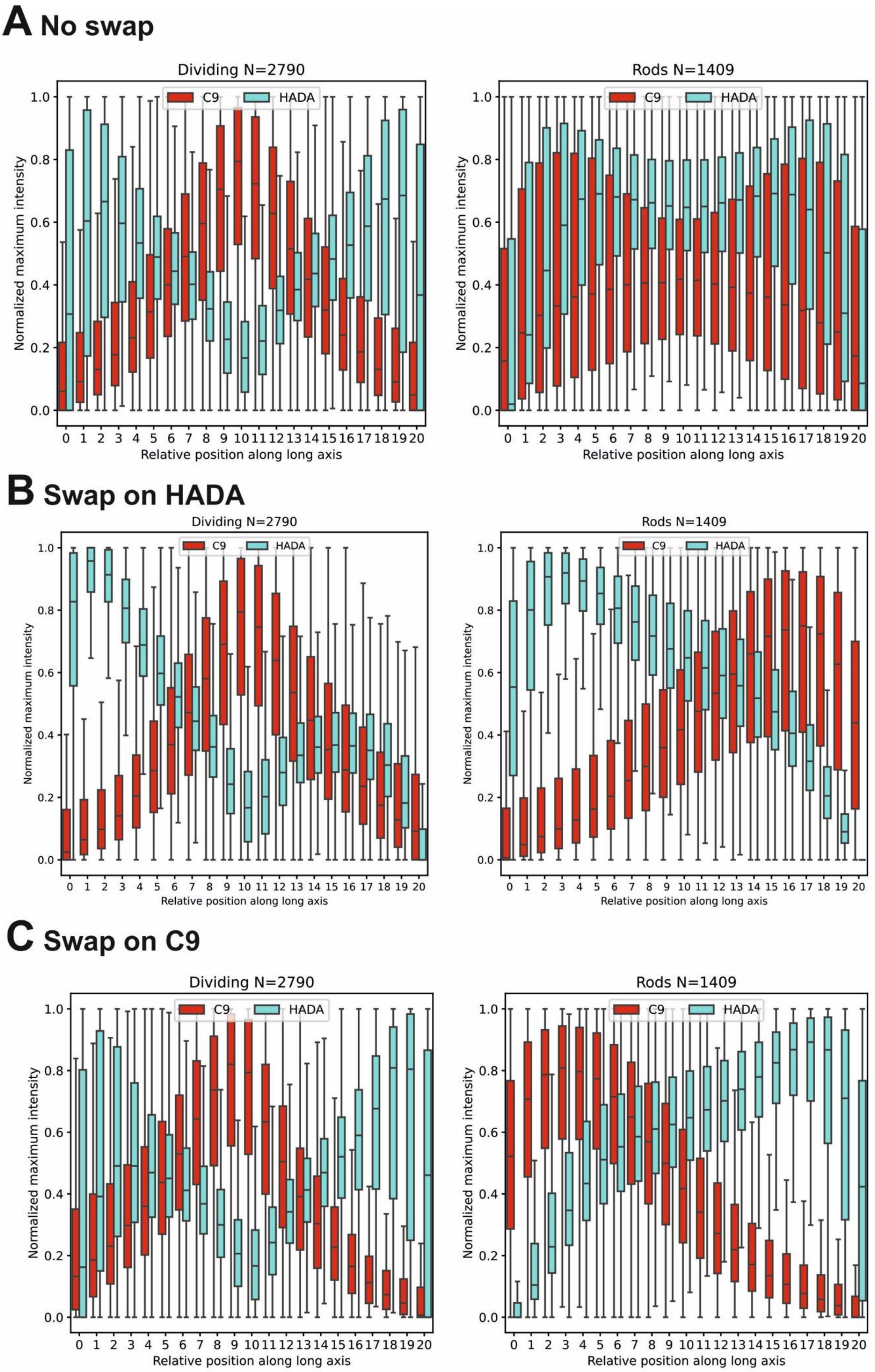
A No swap
Dividing N=2790
Rods N=1409
C9
HADA
Normalized maximum intensity
Relative position along long axis
B Swap on HADA
Dividing N=2790
Rods N=1409
C9
HADA
Normalized maximum intensity
Relative position along long axis
C Swap on C9
Dividing N=2790
Rods N=1409
C9
HADA
Normalized maximum intensity
Relative position along long axis

Appendix Figure S11 - Comparison of channel swapping

A Bacteria can be oriented arbitrarily, but for quantification of fluorescence intensity profiles along the long axis, a starting point must be defined. This is typically done by selecting a fluorescent channel and "swapping" the orientation: if the right half shows a higher intensity than the left half, the cell is flipped so that the higher signal is always positioned on the left. However, this procedure can introduce asymmetry, since the brighter side is systematically aligned to the left. Without swapping, the HADA signal is symmetrically distributed at the poles of dividing cells, and rod-shaped bacteria show no enrichment of either C9 or HADA at the poles.

B With swapping based on the HADA channel (as in Fig. 3D), dividing bacteria display an asymmetric HADA distribution which can be explained as a left-sided bias due to swapping of the HADA channel.

C With swapping based on the C9 channel, the intensity profile in rod-shaped bacteria is nearly identical to (**B**), but inverted. In dividing bacteria, the HADA distribution appears more symmetrical.
