# Supplementary material for: Bactericidal membrane attack complex formation initiates at the new pole of E. coli
Source: EMBO Rep. 2025 Dec 8;27(2):533–54. doi: 10.1038/s44319-025-00669-1 (PMC12852941; doi:10.1038/s44319-025-00669-1)
Supplement: Supplementary file 3 — Movie EV1 [file 44319_2025_669_MOESM3_ESM.zip › Movie EV1/Movie EV1 legend.docx]

**Movie EV1: Time-lapse imaging of E. coli exposed to MAC for 0 minutes.** E. coli MG1655 was pre-incubated with C5-depleted serum. After washing, bacteria were applied to an agar pad with Sytox Blue and imaged every 5-10 minutes for 2.5 hours (Fig. 4). C9-AF647 is shown in red and Sytox in blue. Time stamp indicates hh:mm. Scale bar, 10 µm.
