# Supplementary material for: Bactericidal membrane attack complex formation initiates at the new pole of E. coli
Source: EMBO Rep. 2025 Dec 8;27(2):533–54. doi: 10.1038/s44319-025-00669-1 (PMC12852941; doi:10.1038/s44319-025-00669-1)
Supplement: Supplementary file 12 — Expanded View Figures [file 44319_2025_669_MOESM12_ESM.pdf]

Expanded View Figures

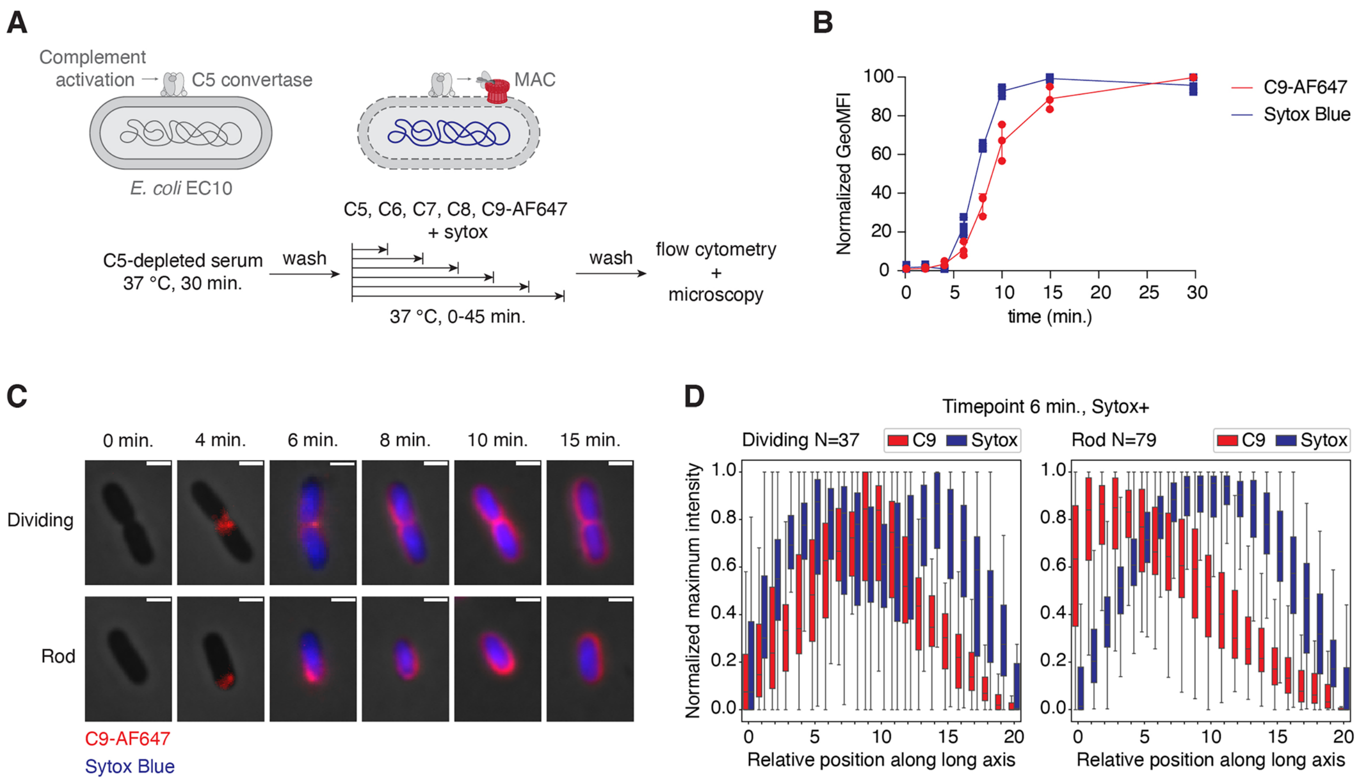

Figure EV1. MAC localization on a clinical *E. coli* isolate.

(**A**) Schematic overview of the experimental setup used to study MAC deposition and inner membrane in time on *E. coli* EC10. (**B**) C9-AF647 deposition and inner membrane damage (Sytox Blue) in time, measured by flow cytometry. (**C**) Phase-contrast and overlayed fluorescence images of selected bacteria at different timepoints. C9-AF647 is shown in red and Sytox in blue. (**D**) Normalized average C9 and Sytox distribution along the long axis of dividing and rod-shaped bacteria after 6 min incubation with C5-C9. Data information: In (**B**), GeoMFI values were normalized by calculating the percentage of the maximum value after log-transformation. Data represent individual values with mean ± SD of three biological replicates. In (**C**), individual bacteria have been chosen to reflect an average bacterium in the sample and are representative for three biological replicates. Scale bars: 2 µm. In (**D**), each box represents the interquartile range (IQR) of the data, with the center line indicating the median relative intensity. The whiskers extend to the most extreme values within 1.5× IQR. Data analysis was performed on all 14 images of one biological replicate, containing 37 dividing and 79 rod-shaped bacteria. Source data are available online for this figure.

© The Author(s)

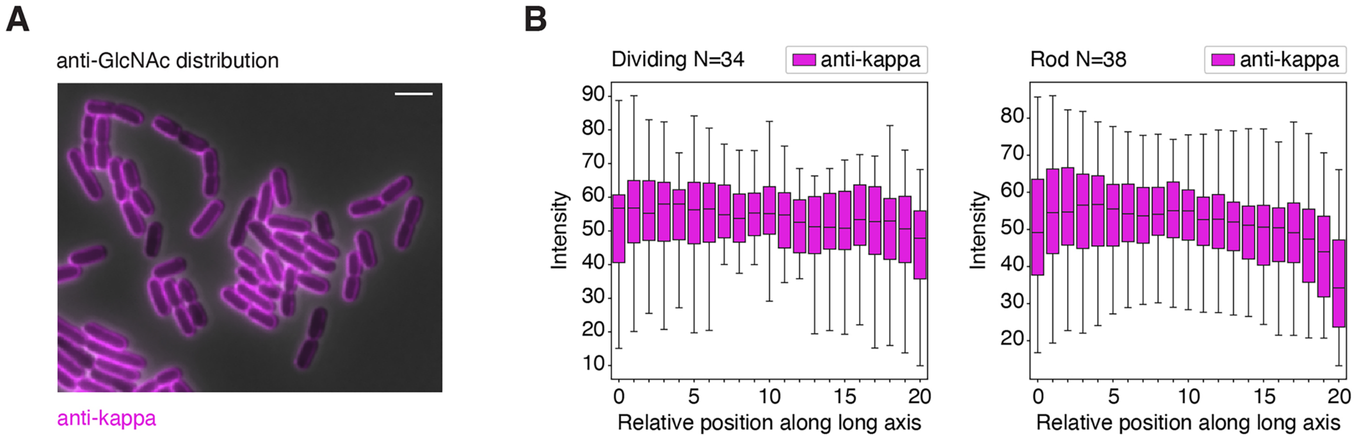

Figure EV2. Anti-GlcNAc IgM distribution on *E. coli.*

(**A**) Phase-contrast and overlayed fluorescence images of bacteria that were incubated with 1 μg/ml anti-GlcNAc IgM followed by staining with a fluorescently labeled anti-kappa F(ab')2 fragment. (**B**) Average anti-GlcNAc IgM distribution along the long axis of dividing and rod-shaped bacteria. Data information: In (**A**), scale bar: 5 μm. In (**B**), each box represents the interquartile range (IQR) of the data, with the center line indicating the median relative intensity. The whiskers extend to the most extreme values within 1.5× IQR. Data analysis was performed on all ten images of one biological replicate, containing 34 dividing and 38 rod-shaped bacteria.

© The Author(s)

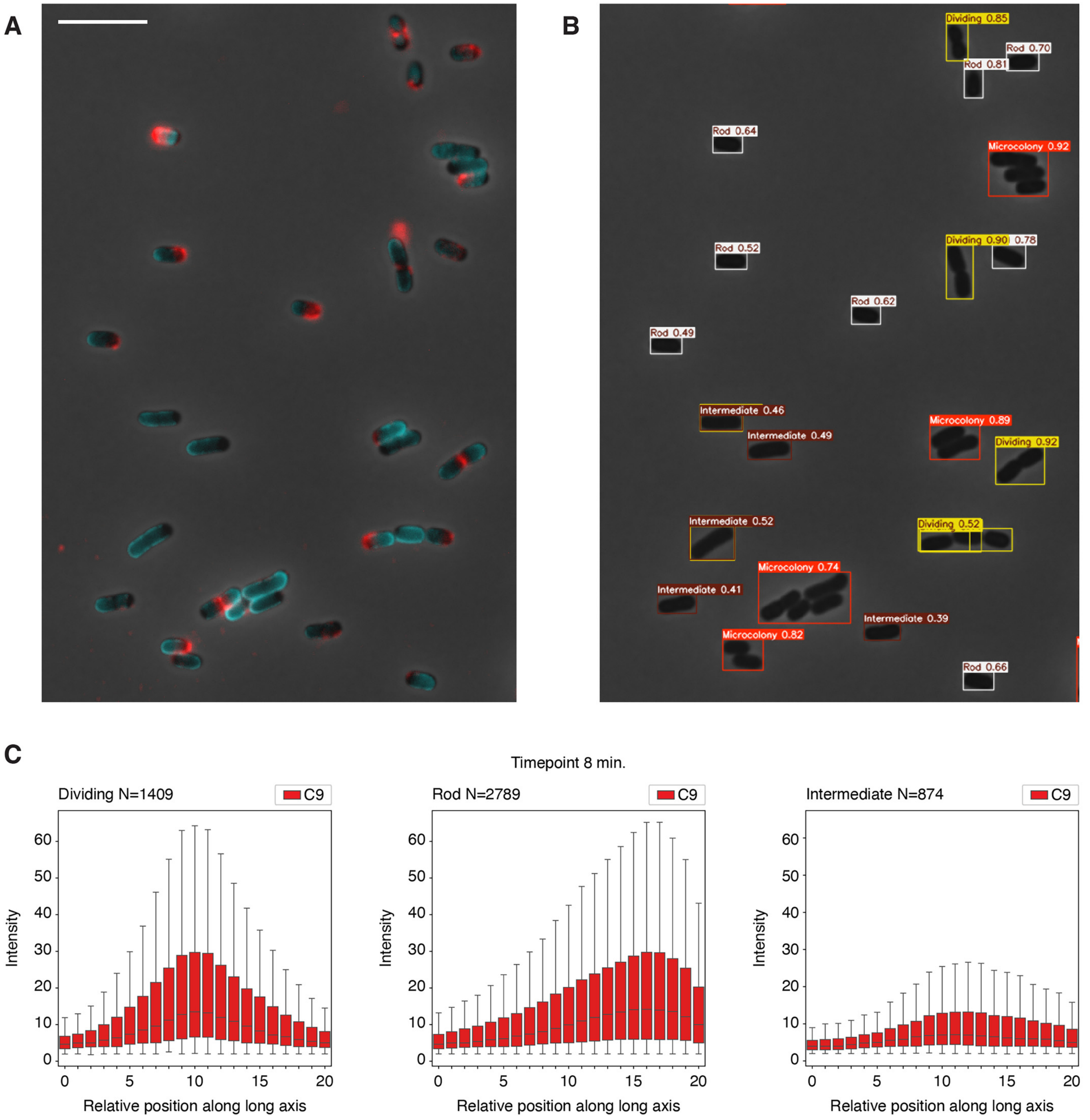

Figure EV3. Analysis of MAC localization on intermediate stage bacteria.

(**A**) Phase-contrast and overlayed fluorescence images of bacteria that were treated as depicted in Fig. 3B. C9-AF647 is shown in red and HADA in cyan. (**B**) Growth stage classification of bacteria shown in (**A**). Dividing bacteria are in yellow boxes, rods in white boxes, intermediates in brown boxes and microcolonies in red boxes. (**C**) Average C9-AF647 distribution along the long axis of dividing, rod-shaped and intermediate stage bacteria. Data information: In (**A**), scale bar: 10 µm. In (**C**), each box represents the interquartile range (IQR) of the data, with the center line indicating the median relative intensity. The whiskers extend to the most extreme values within 1.5× IQR. Data analysis was performed on all 50 images of one biological replicate.

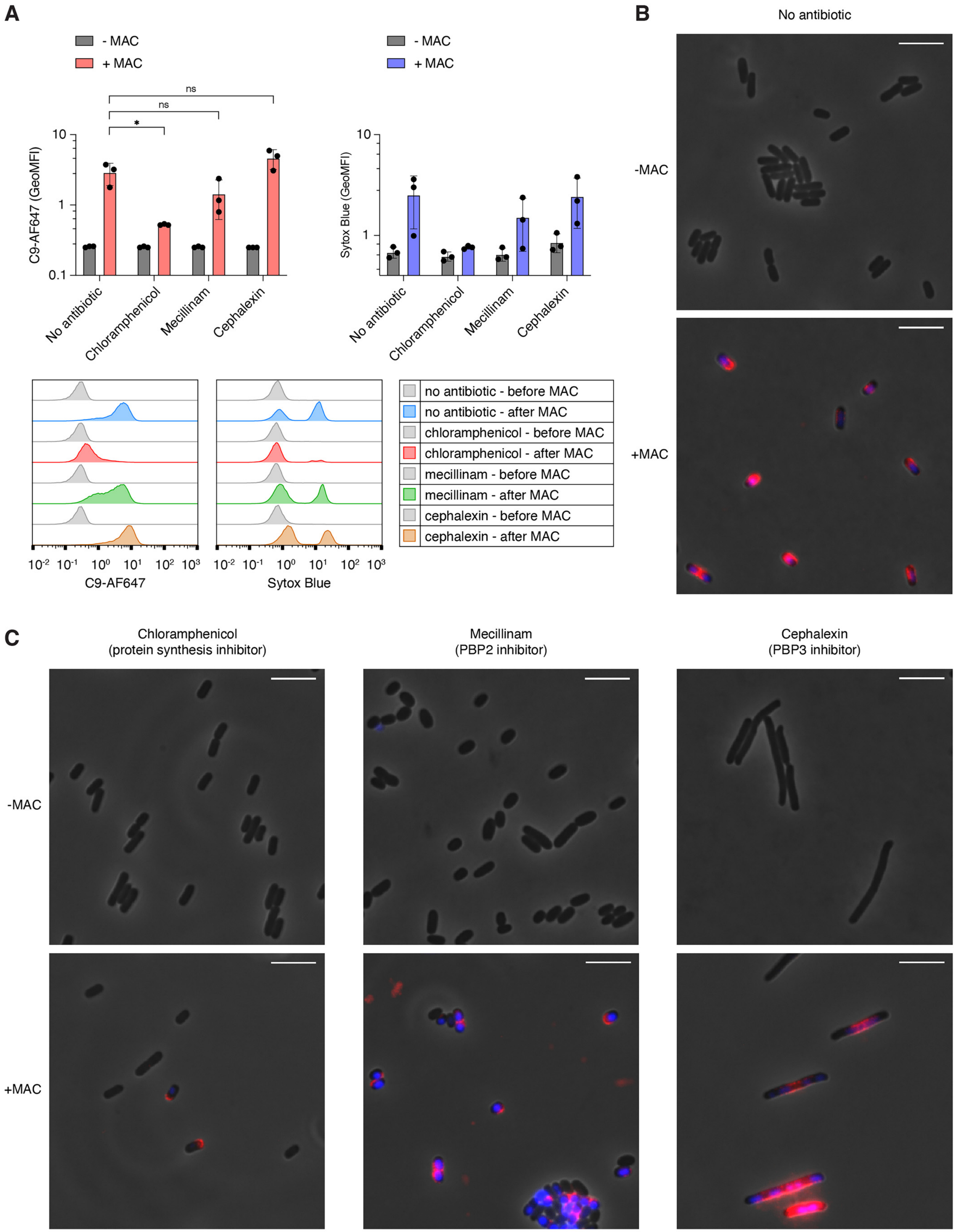
A
- MAC
+ MAC
ns
ns
*
C9-AF647 (GeoMFI)
Sytox Blue (GeoMFI)
No antibiotic
Chloramphenicol
Mecillinam
Cephalexin
no antibiotic - before MAC
no antibiotic - after MAC
chloramphenicol - before MAC
chloramphenicol - after MAC
mecillinam - before MAC
mecillinam - after MAC
cephalexin - before MAC
cephalexin - after MAC
C9-AF647
Sytox Blue
B
No antibiotic
-MAC
+MAC
C
Chloramphenicol
(protein synthesis inhibitor)
Mecillinam
(PBP2 inhibitor)
Cephalexin
(PBP3 inhibitor)

 © The Author(s)

Figure EV4. MAC deposition on antibiotic-treated *E. coli.*

(**A**) *E. coli* MG1655 was pre-incubated with C5-depleted serum, washed, and then incubated with purified MAC components for 8 min. Chloramphenicol, mecillinam or cephalexin were present during incubation with C5-depleted serum and during incubation with purified MAC components. Graphs show flow cytometry analysis of C9-AF647 and Sytox Blue fluorescence intensities before and after incubation with MAC components. (**B**, **C**) Widefield fluorescence images show bacteria before and after incubation with purified MAC components. C9-AF647 is shown in red and Sytox in blue. Data information: In (**A**), data represent individual values with mean ± SD of three biological replicates. Statistics was performed using a one-way ANOVA ($P = 0.0041$ for C9, $P = 0.1889$ for Sytox) followed by Dunnett's post-hoc test, comparing each group with the no antibiotic control. *$P < 0.05$. In (**B**, **C**), images are representative for three biological replicates. Scale bars: 10 µm. Source data are available online for this figure.

© The Author(s)

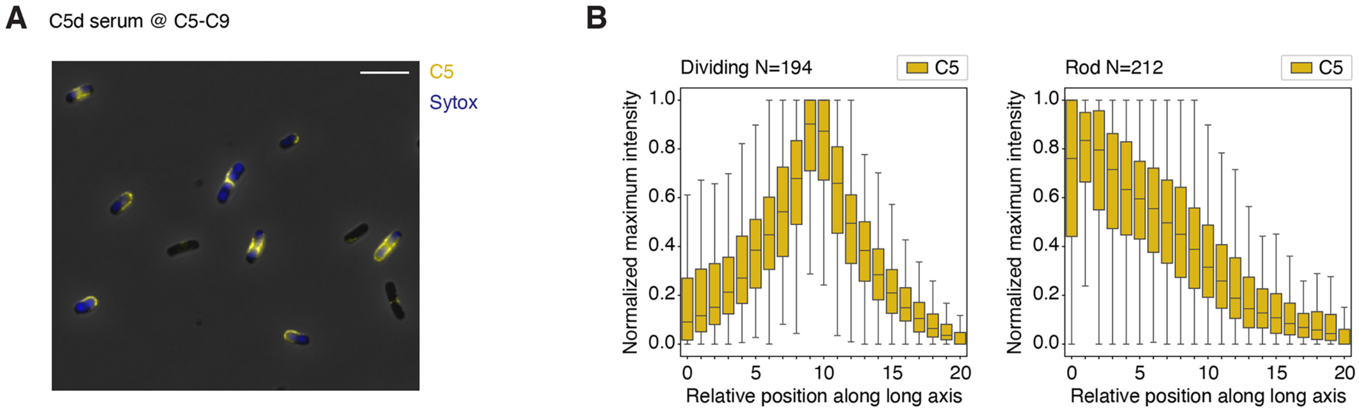

Figure EV5. MAC component C5 also localizes at the new pole of *E. coli*.

(**A**) Convertase-labeled *E. coli* bacteria were exposed to C5-C9 for 4 min followed by staining with anti-C5 and a fluorescently labeled secondary antibody. Localization of C5 (yellow) was determined by phase-contrast and widefield fluorescence microscopy. (**B**) Data analysis of the sample shown in (**A**). Graphs show the normalized average C5 distribution along the long axis of dividing and rod-shaped bacteria. Data information: In (**A**), scale bar: 10 µm. In (**B**), each box represents the interquartile range (IQR) of the data, with the center line indicating the median relative intensity. The whiskers extend to the most extreme values within 1.5× IQR. Data analysis was performed on all 30 images of one biological replicate, containing 194 dividing and 212 rod-shaped bacteria. The image and graphs are representative for three biological replicates.

© The Author(s)
